# Mental health problem recognition and stigma among emerging adults: Findings from the Voices for Well-Being Study (Project VOW)

**DOI:** 10.1007/s00127-026-03207-z

**Published:** 2026-08-28

**Authors:** Annie B. Fox, Jean-Phillipe Laurenceau, E. Carly Hill, Valerie A. Earnshaw

**Affiliations:** 1 School of Health and Rehabilitation Sciences, Mass General Brigham University of Health Professions, Boston, MA USA; 2 Department of Psychological and Brain Sciences, University of Delaware, Newark, DE USA; 3 Department of Human Development and Family Sciences, University of Delaware, Newark, DE USA; 4 Mass General Brigham University of Health Professions, 36 1st Avenue, 02129 Boston, MA USA

**Keywords:** Mental health problem, Mental illness stigma, Modified labeling theory, Emerging adults

## Abstract

**Purpose:**

Emerging adults experience the highest rates of mental health problems but the lowest rates of treatment engagement among U.S. adults. Stigma is a well-known barrier to care, undermining outcomes along the mental health treatment cascade from symptom recognition to treatment seeking. In the present study, we examine differences in experiences of stigma among emerging adults based on the presence, recognition, and/or diagnosis of mental health problems.

**Method:**

The Voices for Well-Being (Project VOW) is a 12-wave longitudinal study of stigma and mental health in emerging adults (ages 18–21) recruited nationally via snowball and convenience sampling, surveyed quarterly for three years. Using baseline data, we classified participants into four groups: (1) no mental health problem history (*n* = 608); (2) recognized and diagnosed (*n* = 2566); (3) recognized and undiagnosed (*n* = 1417) and (4) unrecognized mental health problem (*n* = 91). We examined demographic profiles and compared groups across stigma mechanisms (stereotypes, prejudice, discrimination, perceived, anticipated, experienced, and internalized stigma) using ANCOVA models.

**Results:**

The Project VOW Cohort is demographically diverse with a high mental health burden; 54.4% of participants reported a recognized and diagnosed mental health problem. Emerging adults with no or unrecognized mental health problems endorsed greater stereotypes, prejudice, and discrimination than those with recognized problems. Individuals with recognized and diagnosed conditions reported more experienced and internalized stigma than those with recognized but undiagnosed or unrecognized problems.

**Conclusion:**

Findings highlight the critical role diagnoses play in shaping how people with mental illness are treated and view themselves, informing stigma intervention efforts for emerging adults.

**Supplementary Information:**

The online version contains supplementary material available at https://doi.org/10.1007/s00127-026-03207-z.

Emerging adults (aged 18–25) experience the highest rates of mental illness but the lowest rates of mental health treatment engagement among adults in the United States (US). Epidemiological data suggest that nearly three-quarters of all mental health problems (MHPs) begin by age 24, making emerging adulthood an important developmental period in which individuals may experience the first onset of a MHP [1–2]. In 2023, emerging adults were estimated to experience a higher prevalence of MHPs overall (33.8% vs. 14.1–29.2% among other adult age groups) and major depressive episodes specifically (17.5% vs. 4.5–10.2%) [3]. Slightly fewer emerging adults with MHPs received treatment compared to other adult age groups (51.0% vs. 52.7–55.6%), but more perceived an unmet need for treatment (36.7% vs. 11.5–25.4%) [3]. Stigma, a social process supported by power that results in social devaluation and discrediting [4], undermines outcomes along the full mental health treatment cascade [5]. For example, stigma may prevent emerging adults from recognizing that they have a MHP, although other factors (e.g., mental health literacy) may also contribute [6]. Among those who have recognized their MHP, stigma may also further undermine access to care [7]. Further, emerging adults may be more vulnerable to stigma than other adults given that they are often navigating new social environments that may expose them to new sources of stigma, and may still be developing coping skills and resilience resources to respond to stigma [8]. Our team is conducting a longitudinal study of stigma, mental health, and treatment engagement among emerging adults in the US. The developmental dynamics of emerging adulthood create a unique context for examining how stigma impacts emerging adults and how it changes over time [8]. Herein, we explore experiences of stigma among emerging adults based on the presence, recognition, and/or diagnosis of MHPs.

Stigma may be associated with the recognition of MHPs among emerging adults. According to the Mental Illness Stigma Framework(MISF), individuals may experience and express the social process of stigma via a series of stigma mechanisms [9]. Based on a systematic review of studies measuring mental illness stigma, the MISF identifies seven different stigma mechanisms, organized by relevance to the perceiver (i.e., someone who stigmatizes others) or the target (i.e., someone experiencing a MHP). Stigma mechanisms relevant to the perceiver include perceived stigma, stereotypes, prejudice, and discrimination. *Perceived stigma* involves perceptions of stigma among others in one’s community or society. *Stereotypes* are beliefs about people with MHPs [10]. *Prejudice* is emotions or affective reactions towards people with MHPs [11]. *Discrimination* is unfair or unjust behaviors towards people with MHPs [12, 13].

People without recognized MHPs may endorse more stereotypes, prejudice, and discrimination towards people with MHPs than people with recognized MHPs [6]. One theoretical explanation for this may be that individuals with unrecognized MHPs may engage in “defensive projection” where they endorse more stigma towards people with MHPs in an attempt to distance themselves from the negative personal characteristics associated with having a MHP [14]. Further, a qualitative synthesis of research on stigma and help-seeking suggests that dissonance between one’s preferred social identify and mental illness stereotypes may contribute to stigma endorsement and label avoidance [15]. In contrast, people with recognized MHPs may be more familiar with the symptoms and experience of having a MHP, and evidence generally suggests that familiarity with mental illness is associated with less stigma towards people with mental illness [16]. Further, people without self-reported mental illness report more mental illness stigma than people with self-reported mental illness [17], and evidence suggests that stigma is a barrier to MHP recognition among people with MHPs [7]; therefore, people with unrecognized MHPs may endorse higher stigma than people with recognized MHPs. In a sample of participants reporting clinically significant levels of depression, anxiety or PTSD, Fox and Earnshaw [18] found that individuals who did not label themselves as having a MHP reported higher levels of stereotypes, prejudice, and discrimination compared to those who did label themselves as having a MHP.

Stigma may be further associated with diagnoses of MHPs among emerging adults. The MISF theorizes that people with MHPs experience stigma via several unique mechanisms, including experienced, anticipated, and internalized stigma [9]. *Experienced stigma* is experiences of discrimination from others in the past or present. *Anticipated stigma* is expecting to experience discrimination from others in the future. Finally, *internalized stigma* includes endorsing prejudice and stereotypes about people with MHPs and applying them to the self. Modified Labeling Theory suggests that receiving a MHP diagnosis of shapes both how individuals view themselves and how they are treated by other people [19]. People with diagnoses of MHPs may experience, anticipate, and internalize more stigma than people without diagnoses of MHPs [18].

The current study examined differences in experiences of stigma mechanisms among emerging adults based on the presence, recognition, and/or diagnosis of a MHP. We hypothesized that participants without a recognized MHP would endorse more discrimination, prejudice, stereotypes and less perceived stigma towards people with MHPs than participants with a recognized MHP. We further hypothesized that participants with a self-reported diagnosed MHP would report greater experienced, anticipated, and internalized mental health stigma than and similar levels of perceived stigma as participants without a diagnosed MHP. Data are drawn from the baseline survey of the Voices for Well-Being study (Project VOW), which is a 12-wave, longitudinal study of stigma, mental health, and treatment engagement among emerging adults (ages 18–21) in the United States.

## Method

Project VOW participants complete surveys every three months for three years. Initial recruitment occurred in two phases: In Phase 1 (October 2022-December 2022), a non-profit health research and consulting organization (HRCO) was contracted to collect a sample of young adults in the US using address-based sampling and then later, a combination of social media recruitment and snowball sampling (referred to as subcohort 1). In Phase 2 (February 2023-December 2023), social media and additional recruitment methods (e.g., snowball sampling, research participant registries) were used to recruit participants. In order to keep data collection schedules aligned (February, May, August, November), participants recruited during Phase 2 were grouped into one of three subcohorts based on date of enrollment (referred to as subcohorts 2–4). Participants were eligible if they resided in the US, were 18–21 years old at study registration, and had access to an internet-capable device.

## Procedures and participants

**Fig. 1:**
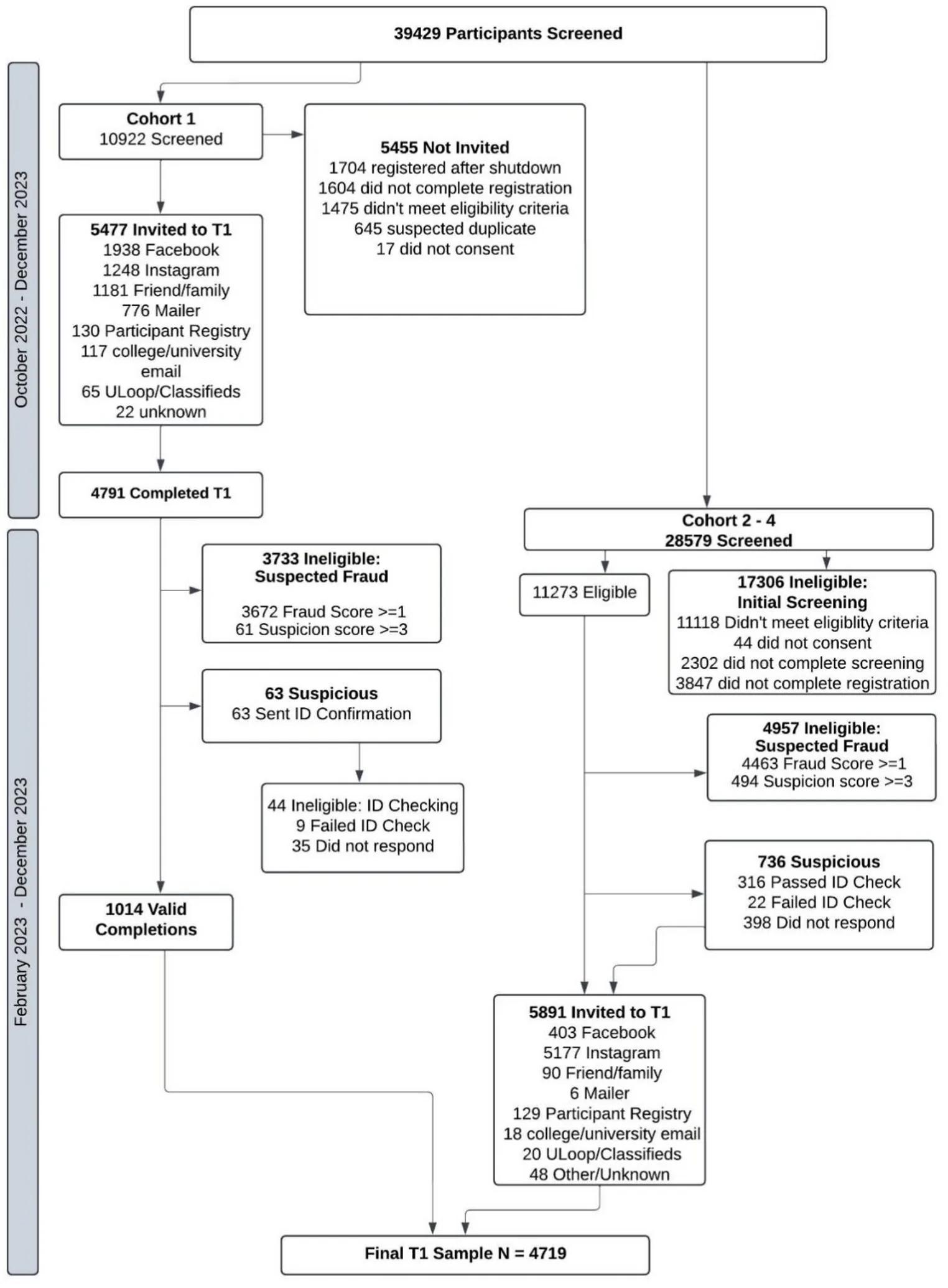
Enrollment diagram for the voices for well-being study

In both recruitment phases, participants completed a registration survey followed by the first survey (referred to as the T1 survey) and fraud checking procedures were applied. Although criteria for fraud checking were the same for both phases, the timing of the fraud checking differed (i.e., with Phase 1 focusing more on T1 data, and Phase 2 focusing more on registration data). Figure 1 includes the enrollment diagram.

### Phase 1 recruitment

The HRCO used address-based sampling to identify an initial sample of 40,500 emerging adults, oversampling for Black individuals (25%). Names and mailing addresses of potential participants were obtained via marketing lists purchased from American Student List (ASL) Marketing, which maintains names and addresses of over five million emerging adults across the US. Potential participants were mailed an informational letter and follow-up postcards with links to the registration site. Participants registered for the study via a screening eligibility questionnaire that included: date of birth, current location, and access to an internet-capable device. Eligible participants were then presented with an online consent form; those who agreed to participate were asked to provide their full contact information and complete a short demographic questionnaire. CAPTCHA and cookies were used to prevent bots and duplicate completions, and IP addresses were collected. Eligible respondents who completed registration received $5 and a T1 survey invitation. Participants who completed T1 were paid $15.

Initial recruitment procedures fell short (*n* = 917 completed T1; 2.3% response rate), necessitating a pivot to online recruitment via social media, additional snowball sampling, and college/university outreach. A series of Meta (Facebook, Instagram) ads was created that targeted 18–21 year olds interested in a “paid research opportunity” around creating an “online community of voices to impact the health and well-being of young adults,” as well as ads that encouraged young adults to “share their voice.” Ads included different taglines, including “More Voices. More Impact. Project VOW,” “Share your voice,” “We need you!” and “Make an impact.” The study website also contained a recruitment video and a similar description of the project.

Online recruitment during Phase 1 lasted approximately two weeks, after which we hit our initial recruitment goal by enrolling an additional 3,874 participants. However, due to abnormalities in the data, a comprehensive fraud protocol was developed and applied to the registration and T1 survey data, resulting in the removal of 3,777 participants . The final Phase 1 sample size was 1,014. Recruitment then moved to Phase 2 with a comprehensive fraud protocol.

### Phase 2 recruitment

Phase 2 took place via social media ads on Meta, snowball sampling, participant registries (e.g., Research Match), and ads on college and university classified ad websites (ULoop) using the Phase 1 study registration procedures. In addition to CAPTCHA and IP addresses, additional fraud indicators were included. Participants were asked to provide their dates of birth at the beginning and end of the eligibility screening, and respond to open-ended questions that allowed us to screen out non-sensical responses (e.g., responses of “blue” to “What is your favorite ice cream?”). The social media link was changed weekly to prevent fraudulent distribution. In total, there were 28,579 registration responses. Respondents who passed an initial screening for eligibility and fraud via email addresses, contact information, and IP addresses were invited to complete the T1 survey (*n* = 5,891). T1 data was also fraud checked using IP addresses and open-response items. A total of 3,705 completed the T1 survey and passed fraud screening. Cohorts from Phases 1 and 2 were then combined (*n* = 4,719). We also compared Phase 1 and 2 participants on demographic and mental health variables; effect sizes were negligible to small (ds = 0.02-0.10, Vs = 0.01 − 0.12), suggesting recruitment method did not result in substantive differences between cohorts.

## Measures

### Demographics

Participants reported their gender identity, sexual orientation, race, ethnicity, educational experience, and disability status. Additional demographics are included in supplemental Table 1.

### Mental health problem history

Participants were asked three yes/no mental health history questions: (a) Have you ever experienced a mental health problem lasting at least two weeks? (b) Was there ever a time when you felt that you might need to see a professional because of problems with your mental health, emotions or nerves, or your use of alcohol or drugs? And (c) Have you ever been diagnosed by a healthcare professional with a mental health condition (e.g., depression, bipolar disorder, generalized anxiety, PTSD, etc.)?

### Depression

Depressive symptoms in the past two weeks were measured with the Patient Health Questionnaire-9 (PHQ-9) [20, 21]. Items were measured using a 4-point scale (0 = never, 1 = several days, 2 = more than half the days, and 3 = Nearly every day). Scores were summed to create a total PHQ score (range 0–27; α = 0.89). Participants with PHQ scores > = 8 were classified as having probable depression [22].

### Generalized anxiety

Anxiety symptoms in the past two weeks were measured with the Generalized Anxiety Disorder Scale-7(GAD-7) [23], using a 4-point scale (0 = not at all, 1 = several days, 2 = more than half the days, and 3 = nearly every day). Scores were summed to create a total GAD score (range 0–21; α = 0.91). Participants with GAD scores > = 8 were classified as having probable depression [24].

### Perceived stigma

The 9-item Perceived Stigma subscale from the Depression Stigma Scale (DSS) [25] measures participants’ perceptions of what other people think about people with mental illness (e.g., “Most people believe that mental illness is a sign of personal weakness”) using a 5-point scale (0 = strongly disagree to 4 = strongly agree; α = 0.87).

### Stereotypes

The 9-item Personal Stigma subscale from the Depression Stigma Scale (DSS) [25] measures participants’ attitudes related to personal stigma (e.g., “People with mental illness are dangerous”) using a 5-point scale (0 = strongly disagree to 4 = strongly agree; α = 0.82).

### Prejudice

The Fear/Avoidance and Malevolence subscales from the Prejudice Towards People with Mental Illness Inventory (PPMI) [26] contain 16 items that measure participants’ attitudes related to prejudice towards people with mental illness (e.g., “People with mental illness do not deserve our sympathy”). Items used a 9-point scale (0 = very strongly disagree to 8 = very strongly agree; α = 0.89).

### Discrimination

The 4-item discrimination subscale from the Reported and Intended Behavior Scale (RIBS) [27] assesses participants’ willingness to discriminate (e.g., “In the future, I would be willing to live with someone with a mental health problem”) using a 5-point scale (0 = strongly disagree, 4 = strongly agree) with a “don’t know” option that is recoded as the neutral response. Scores were summed to create a composite (score range 0–20; α = 0.89).

### Experienced stigma

The 22-item Discrimination and Stigma Scale (DISC) [28] measures how often people experience discrimination because of their mental illness (e.g., “Have you been treated unfairly in making or keeping friends? ) using a 4-point scale (0 = not at all to 3 = a lot; α = 0.92).

## Anticipated stigma

The 14-item Questionnaire on Anticipated Discrimination scale (QUAD) [29] assesses the extent to which people with MHPs anticipate that they will personally experience discrimination across a range of contexts (e.g., “If employers know I have a mental health problem, they will treatment me unfairly”) using a 4-point scale (0 = strongly disagree to 3 = strongly agree; α = 0.88).

### Internalized stigma

The 6-item alienation subscale of the Internalized Stigma of Mental Illness (ISMI) scale [30] measures the degree to which respondents internalize attitudes towards mental illness (e.g., “I feel out of place in the world because I have a mental illness”) using a 4-point scale (0 = strongly disagree to 3 = strongly agree; α = 0.84).

### Data analysis

Participants were classified into groups based on presence, recognition, and/or diagnosis of a current or lifetime MHP. Participants who self-reported they had never experienced a MHP, never received professional help, had never been diagnosed with a MHP, and did not meet cut-offs (scores > = 8) for current depression or anxiety were classified into the “no mental health problem history” group (*n* = 608). Those who self-reported they had ever experienced a mental health problem or received professional help and had received a diagnosis were classified “recognized and diagnosed mental health problem” (*n* = 2566). Participants who experienced a MHP but had not received a diagnosis were classified as “recognized and undiagnosed mental health problem” (*n* = 1417). Individuals who had never experienced a MHP or sought professional help but whose current GAD or PHQ scores exceeded the clinical cutoffs were classified as “unrecognized mental health problem” (*n* = 91). A small number of participants (*n* = 37) reported they had never experienced a MHP but had received a diagnosis at some point in their lifetime. Given the small number of participants in this group (0.8% of overall sample) and the lack of clarity around what it means for participants to report a diagnosis but deny ever experiencing a MHP, we excluded this group from primary analyses but included their demographics in Table 1.

**Table 1:** Socio-demographic and mental health characteristics of the VOW cohort by mental health status

	Full Sample	No MHP	Unrecognized MHP	Recognized MHP/No DX	Recognized MHP/DX	Unrecognized DX	*p*-value
	*N* = 4719 (100%)	*N* = 608 (12.88%)	*N* = 91 (1.93%)	*N* = 1417 (30.03%)	*N* = 2566 (54.38%)	*N* = 37 (0.78%)	
Age, M (SD)	19.95 (1.14)	19.83 (1.11)	19.63 (1.13)	19.85 (1.12)	20.05 (1.14)	19.72 (0.98)	< 0.001
Gender Identity							< 0.001
Man	1189 (25.21%)	304 (50.08%)	40 (43.96%)	382 (26.96%)	455 (17.75%)	8 (21.62%)	
Woman	2503 (53.07%)	296 (48.76%)	48 (52.75%)	840 (59.28%)	1293 (50.43%)	26 (70.27%)	
Gender Diverse	1024 (21.71%)	7 (1.15%)	3 (3.30%)	195 (13.76%)	816 (31.83%)	3 (8.11%)	
Sexual Orientation							< 0.001
Gay	245 (5.20%)	16 (2.64%)	3 (3.30%)	56 (3.96%)	170 (6.63%)	0 (0.00%)	
Lesbian	325 (6.89%)	10 (1.65%)	4 (4.40%)	60 (4.24%)	249 (9.71%)	2 (5.41%)	
Straight	1920 (40.73%)	500 (82.37%)	69 (75.82%)	669 (47.31%)	659 (25.69%)	23 (62.16%)	
Bisexual	1466 (31.10%)	61 (10.05%)	10 (10.99%)	424 (29.99%)	964 (37.58%)	7 (18.92%)	
Something else	758 (16.08%)	20 (3.29%)	5 (5.49%)	205 (14.50%)	523 (20.39%)	5 (13.51%)	
Race							< 0.001
Native American/Alaskan	71 (1.51%)	2 (0.33%)	2 (2.20%)	34 (2.40%)	33 (1.29%)	0 (0.00%)	
Black	687 (14.59%)	126 (20.86%)	25 (27.47%)	240 (16.97%)	289 (11.28%)	7 (18.92%)	
Asian	414 (8.79%)	89 (14.74%)	23 (25.27%)	174 (12.31%)	125 (4.88%)	3 (8.11%)	
Filipino	58 (1.23%)	10 (1.66%)	1 (1.10%)	26 (1.84%)	21 (0.82%)	0 (0.00%)	
West Asian/Middle Eastern/North African	47 (1.00%)	11 (1.82%)	1 (1.10%)	13 (0.92%)	22 (0.86%)	0 (0.00%)	
Native Hawaiian	6 (0.13%)	1 (0.17%)	1 (1.10%)	2 (0.14%)	2 (0.08%)	0 (0.00%)	
Other Pacific Islander	7 (0.15%)	1 (0.17%)	0 (0.00%)	3 (0.21%)	3 (0.12%)	0 (0.00%)	
White/European	2900 (61.60%)	315 (52.15%)	27 (29.67%)	745 (52.69%)	1789 (69.83%)	24 (64.86%)	
Other	356 (7.56%)	35 (5.79%)	4 (4.40%)	120 (8.49%)	196 (7.65%)	1 (2.70%)	
Multi-racial	162 (3.44%)	14 (2.32%)	7 (7.69%)	57 (4.03%)	82 (3.20%)	2 (5.41%)	
Hispanic	884 (18.85%)	118 (19.54%)	14 (15.38%)	322 (22.85%)	424 (16.63%)	6 (16.67%)	0.006
Employment Status							< 0.001
Full-time paid employment	418 (8.88%)	44 (7.28%)	3 (3.33%)	104 (7.34%)	265 (10.35%)	2 (5.41%)	
Part-time paid employment	2227 (47.29%)	283 (46.85%)	36 (40.00%)	648 (45.73%)	1243 (48.54%)	17 (45.95%)	
Voluntary work (unpaid)	159 (3.38%)	19 (3.15%)	3 (3.33%)	56 (3.95%)	79 (3.08%)	2 (5.41%)	
Not employed, looking	1245 (26.44%)	134 (22.19%)	32 (35.56%)	422 (29.78%)	647 (25.26%)	10 (27.03%)	
Not employed, not looking	660 (14.02%)	124 (20.53%)	16 (17.78%)	187 (13.20%)	327 (12.77%)	6 (16.22%)	
Current Educational Setting							< 0.001
Not currently enrolled	762 (16.43%)	52 (8.81%)	12 (13.95%)	182 (13.08%)	508 (20.06%)	8 (21.62%)	
High school	456 (9.83%)	66 (11.19%)	21 (24.42%)	138 (9.92%)	227 (8.96%)	4 (10.81%)	
College or university	3130 (67.50%)	441 (74.75%)	49 (56.98%)	995 (71.53%)	1626 (64.19%)	19 (51.35%)	
Fully online college/university	159 (3.43%)	16 (2.71%)	3 (3.49%)	43 (3.09%)	93 (3.67%)	4 (10.81%)	
Vocational/trade school	39 (0.84%)	5 (0.85%)	0 (0.00%)	12 (0.86%)	22 (0.87%)	0 (0.00%)	
Other educational setting	65 (1.40%)	6 (1.02%)	1 (1.16%)	16 (1.15%)	41 (1.62%)	1 (2.70%)	
More than one setting	26 (0.56%)	4 (0.68%)	0 (0.00%)	5 (0.36%)	16 (0.63%)	1 (2.70%)	
Mental Health Symptoms							
PHQ, sum (SD)	10.40 (6.59)	2.89 (2.26)	10.80 (3.88)	9.87 (5.94)	12.40 (6.35)	6.00 (5.45)	< 0.001
GAD, sum (SD)	8.84 (5.85)	2.04 (2.22)	9.15 (3.88)	8.28 (5.23)	10.80 (5.51)	4.59 (5.06)	< 0.001
Mental Health Diagnoses							
Generalized anxiety disorder	2022 (42.85%)				2005 (78.14%)	17 (45.95%)	
Depression	2016 (42.72%)				1997 (77.83%)	19 (51.35%)	
Post-traumatic stress disorder	625 (13.24%)				621 (24.20%)	4 (10.81%)	
Eating disorder	434 (9.20%)				431 (16.80%)	3 (8.11%)	
Obsessive compulsive disorder	358 (7.59%)				355 (13.83%)	3 (8.11%)	
Social phobia	292 (6.19%)				289 (11.26%)	3 (8.11%)	
Other	262 (5.55%)				259 (10.09%)	3 (8.11%)	
Borderline personality disorder	163 (3.45%)				162 (6.31%)	1 (2.70%)	
Bipolar disorder II	120 (2.54%)				119 (4.64%)	1 (2.70%)	
Bipolar disorder I	110 (2.33%)				110 (4.29%)	0 (0.00%)	
Panic disorder	104 (2.20%)				102 (3.98%)	2 (5.41%)	
Schizophrenia	19 (0.40%)				19 (0.74%)	0 (0.00%)	
Multiple Diagnoses	1978 (76.67%)				1962 (77.03%)	16 (48.48%)	
Mental Health Treatment (lifetime)	2891 (61.33%)	23 (3.79%)	4 (4.40%)	505 (35.66%)	2338 (91.22%)	21 (56.76%)	< 0.001

Note. n% unless otherwise indicated; MHP = mental health problem, DX = diagnosis

Descriptive statistics were calculated for the overall sample and by mental health history group. One-way ANCOVAs were used to examine differences in stigma mechanism scores by mental health history group. Analyses controlled for gender identity, race, and family income. Significant ANCOVAs were followed up with pairwise comparisons using Cohen’s *d* for effect size. Analyses were conducted in R; effect sizes were calculated using the *emmeans* package [31].

## Results

### Participant overview

Key participant characteristics are included in Table 1; an expanded list of socio-demographics is included in Supplemental Table 1. In total, *n* = 4,719 participants completed the baseline survey. A minority of participants (12.9%) had no MHP history. Over half of participants (54.4%) had a recognized and diagnosed MHP, 30.0% had a recognized and undiagnosed MHP, and 1.9% had an unrecognized and undiagnosed current MHP.

On average, participants were *M* = 20.0 (*SD* = 1.14) years old. Over half of participants (53.1%) identified as women, 25.2% identified as men, and 21.7% identified as gender diverse. There were higher percentages of gender diverse participants in the recognized and diagnosed MHP and recognized and undiagnosed MHP groups than in the unrecognized MHP and no MHP groups. Most participants identified as straight (40.7%) or bisexual (31.1%), with fewer identifying as lesbian (6.9%), gay (5.2%), or something else (16.1%). Higher percentages of participants identified as straight in the no MHP and unrecognized MHP groups, and higher percentages of participants identified as sexual minorities in the recognized and diagnosed MHP and recognized and undiagnosed MHP groups. Regarding race, 61.6% of participants identified as White/European, 14.6% identified as Black, and 8.8% identified as Asian. Fewer White/European and more Black and Asian participants had an unrecognized MHP. Regarding ethnicity, 18.9% of participants identified as Hispanic. Close to half (47.3%) of participants reported part-time paid employment, 26.3% reported not being employed and looking, 14.0% reported not being employed and not looking, 8.9% reported full-time paid employment, and 3.4% reported voluntary unpaid work. Over half (67.5%) of participants were enrolled in college or a university, 16.4% were not currently enrolled in an educational setting, and 9.8% were in high school.

On average, participants reported mild to moderate mental health symptoms and a high rate of mental health diagnoses. Across the full sample, average scores on the PHQ were 10.40 (*SD* = 6.59), and 8.84 (*SD* = 5.85) for the GAD-7. Symptom scores were highest for participants in the recognized and diagnosed group, followed by the unrecognized group, and then recognized with no diagnosis group. The no MHP group had the lowest depression and anxiety scores. GAD and depression were the most commonly reported mental health diagnoses (42.9% and 42.7%, respectively), followed by post-traumatic stress disorder (13.2%), eating disorder (9.2%), obsessive compulsive disorder (7.6%), and social phobia (6.19%). Many participants (76.7%) reported multiple diagnoses.

## Differences in stigma mechanisms

Table 2 contains the results of the ANCOVA models. Given the large sample size, we interpret effect sizes instead of p-values. Participants with no MHPs reported less perceived stigma than participants with an unrecognized MHP, a recognized and undiagnosed MHP, or a recognized and diagnosed MHP. Effect sizes were small to moderate (*d*s < = 0.44). Differences in perceived stigma between participants in other groups were negligible.

**Table 2:** Descriptive statistics, model results, and effect sizes by mental health status group

	No MHP (1)		Unrecognized MHP (2)		Recognized MHP/No DX (3)		Recognized MHP/DX (4)			Pairwise Comparisons-Effect Sizes (Cohen’s d)					
	*N* = 608		*N* = 91		*N* = 1417		*N* = 2566								
	***M***	***SD***	***M***	***SD***	***M***	***SD***	***M***	***SD***	***F***	1 v 2	1 v 3	1 v 4	2 v 3	2 v 4	3 v 4
Perceived stigma	2.04	0.74	2.37	0.82	2.32	0.74	2.39	0.72	36.05^***^	-0.40	-0.35	-0.44	0.05	-0.05	-0.10
Stereotypes	1.07	0.64	1.14	0.65	0.77	0.52	0.50	0.42	302.72^***^	-0.10	0.51	0.95	0.62	1.05	0.43
Prejudice	3.43	1.05	3.47	1.27	2.67	1.01	2.14	0.88	394.27^***^	-0.03	0.70	1.10	0.67	1.07	0.40
Discrimination	0.91	0.85	1.05	0.92	0.52	0.69	0.29	0.59	179.06^***^	-0.12	0.54	0.78	0.65	0.89	0.24
Experienced stigma			0.43	0.56	0.38	0.42	0.66	0.53	112.70^***^				0.08	-0.42	-0.50
Anticipated stigma			1.21	0.62	1.20	0.53	1.25	0.55	4.96^**^				-0.00	-0.06	-0.06
Internalized stigma			0.97	0.67	1.22	0.69	1.49	0.69	73.83^***^				-0.37	-0.72	-0.35

Note. ^***^p<.001; ^**^p<.01; MHP = mental health problem, DX = diagnosis. Analyses controlled for gender identity, race, and family income

Participants with no MHPs and unrecognized MHPs reported the highest levels of discrimination, stereotypes, and prejudice. Although discrimination, stereotypes, and prejudice means were slightly higher among participants with unrecognized MHPs, effect size estimates suggested that these differences were negligible (*d*s < = 0.20). Differences between participants with no MHPs and unrecognized MHPs versus participants with recognized diagnosed or undiagnosed MHPs were medium to large in effect size. Participants with no MHPs and unrecognized MHPs reported greater discrimination, stereotypes, and prejudice than participants with recognized and diagnosed or undiagnosed MHPs. Finally, differences between participants with recognized and undiagnosed MHPs versus participants with recognized and diagnosed MHPs were small to medium in effect size. Participants with recognized and undiagnosed MHPs reported greater discrimination, stereotypes, and prejudice than participants with recognized and diagnosed MHPs.

Participants with a recognized and diagnosed MHP reported the greatest experienced and internalized stigma. Differences between participants with a recognized and diagnosed MHP and participants with a recognized and undiagnosed MHP or an unrecognized MHP were generally small to medium in effect size. The largest observed difference was in internalized stigma between participants with a recognized and diagnosed MHP and participants with an unrecognized MHP. Differences between participants with a recognized and undiagnosed MHP and participants with an unrecognized MHP were negligible in effect size for experienced stigma but small in effect size for internalized stigma. Differences in anticipated stigma between participants in all three groups were negligible in effect size.

## Discussion

The current study explored differences in experiences of stigma mechanisms identified by the MISF among emerging adults based on the presence, recognition, and/or diagnosis of a MHP. Emerging adults with no MHPs or unrecognized MHPs endorsed greater stereotypes, prejudice, and discrimination than those with recognized MHPs (including those having a diagnosed or undiagnosed MHP). Among emerging adults with MHPs, those with recognized and diagnosed MHPs reported more experienced and internalized stigma than those with either recognized but undiagnosed MHPs or those with unrecognized MHPs.

Findings draw attention to the key role that diagnoses may play in shaping how people with mental illness are treated by others and view themselves, which is consistent with Modified Labeling Theory [19]. In the present study, individuals with recognized and diagnosed MHPs were more likely to report experienced and internalized stigma, providing support to the theory that diagnostic labels may trigger negative social responses from others, which in turn may negatively impact people with MHPs. This finding is also consistent with the “insight paradox” where greater insight into one’s illness is associated with increased self-stigma [32], and aligns with work that suggests that not self-labeling may be a protective strategy to avoid the negative effects of stigma [18]. A systematic review of vignette-based experimental studies of diagnostic labeling found more studies demonstrating negative effects of diagnostic labeling versus studies showing positive or neutral effects [33]. Nevertheless, the diagnosed and recognized group had the highest depression and anxiety symptom burden, and nearly every participant had received treatment at some point in their lifetimes (91%). It is notable, however, that analyses in the present study were cross-sectional and derived from baseline data from the VOW cohort. In the future, we will be able to use the longitudinal VOW data to better understand the temporal relationships between labeling, stigma, and treatment seeking.

The demographic differences between groups were largely consistent with existing literature. Gender and sexually diverse participants were more likely to be in the recognized groups, consistent with the well-documented rates of elevated MHPs in these populations [34, 35]. With respect to race, fewer White participants and more Black and Asian participants were concentrated in the unrecognized group. This is consistent with literature documenting racial disparities in MHP recognition and treatment-seeking [36, 37] and may reflect racial or cultural differences in mental health literacy, structural barriers to care, and increased mental illness stigma experienced within these communities [38, 39].

This study is grounded in two complementary theoretical frameworks: modified labeling theory and the MISF, both of which acknowledge that stigma applies to both the stigmatizer and the stigmatized. However, it is important to note that the boundaries between stigma mechanisms that apply to the stigmatizer and the stigmatized are fluid; an individual with a MHP can experience, anticipate, or internalized stigma, while simultaneously holding negative stereotypes or discriminating against others with MHPs. Further, these stigma mechanisms may be additive or interactive, or occur in a process that unfolds over time.

The Project VOW cohort was recruited to examine the longitudinal relationships between stigma and MHPs in young adults in order to better target stigma interventions and improve treatment seeking. Baseline characteristics of the cohort indicate that Project VOW participants are demographically diverse with a high mental health burden. Across the sample, 54.4% of participants reported a recognized and diagnosed MHP, with the most common diagnoses being generalized anxiety disorder (42.8%) and depression (42.7%). These rates are higher than those estimated for emerging adults using recent nationally representative surveys. More participants recognized that they had a MHP that was undiagnosed (30.0%) or had an unrecognized MHP (1.9%). Higher rates of MHPs within this cohort may be due to a combination of factors. For example, participants were recruited from social media and evidence suggests both that social media usage is associated with worse mental health among emerging adults [40] and that people with MHPs may be more likely to use social media to connect with others [41]. The Project VOW cohort also includes high percentages of sexual minority (59.3%) and gender diverse (21.7%) participants, and sexual minority and gender diverse young people tend to experience worse mental health than their peers [42].

There are several reasons why the cohort may have high percentages of sexual minority and gender diverse (SGD) emerging adults. Some research suggests that SGD youth may spend more time on social media than their peers [43], and much of the sample was recruited from social media. Second, younger adults are more likely to identify as SGD. Recent work suggests that 22.3% of Generation Z identify as lesbian, gay, bisexual, transgender, or something other than heterosexual in comparison to 9.8% of millennials, 4.5% of Generation X, 2.3% of baby boomers, and 1.1% of the Silent Generation [44]. Third, recent work suggests that self-report gender identification is sensitive to measurement variation (including question wording and available response options) [45, 46]. It is possible that our approach to gender measurement, which allowed participants to identify as a man, woman, transgender, non-binary, or none of these, may contribute to the higher percentage of gender diverse participants in our sample. The Project VOW cohort is comparable to the U.S. adult population in terms of percentage of adults identifying as Hispanic (18.9% vs. 19.5%) and Black (14.6% vs. 13.7%), and has a slightly lower percentage of participants identifying as White (61.6% vs. 75.3%) [47]. This cohort affords a unique opportunity to examine differences in and eventually trajectories of experiences of stigma associated with MHPs.

## Limitations and future directions

Although the Project VOW cohort is relatively diverse, it is not representative of the U.S. emerging adult population. Men are underrepresented in the cohort, and most participants were recruited via social media. The underrepresentation of men is consistent with broader patterns in mental health research, where men are less likely to seek care [48] or participate in research, particularly via social media recruitment [49, 50]. Advertising the study as examining “well-being” may have attracted individuals who were interested in sharing about their mental health, potentially contributing to the higher rates of MHPs. Additionally, self-reported mental health histories may be impacted by recall or social desirability biases. This study leveraged baseline data to examine cross-sectional differences based on the presence, recognition, and/or diagnosis of a MHP. Future analyses can use Project VOW’s longitudinal data to explore treatment-related outcomes and temporal changes in stigma over time during this critical time period.

## Conclusions

Emerging adults experience high rates of MHPs and low rates of mental health treatment engagement [3], and may be particularly vulnerable to stigma as a treatment barrier [8]. Results can inform intervention efforts to address stigma among emerging adults. Reducing stereotypes, prejudice, and discrimination among emerging adults without MHPs and without recognized MHPs may decrease structural stigma and barriers to recognizing MHPs [7]. It may also be important to address experienced and internalized stigma among emerging adults with diagnosed MHPs. Similar to what has been tested with sexual and gender minority populations [51], it may be possible to adapt mental health treatment to address these experiences of stigma. Finally, interventions to eliminate stigma within environments are critically important to prevent exposure to stigma among emerging adults with MHPs [5].

## Supplementary Information

Below is the link to the electronic supplementary material.

Supplementary Material 1

## Data Availability

The data supporting the findings of this study are available in the NIMH Data Archive (NDA), [Collection ID 4224]. Access to the data can be requested through the NDA Data Access Committee and is subject to the Data Use Certification (DUC).
