# Supplementary material for: Mental health problem recognition and stigma among emerging adults: Findings from the Voices for Well-Being Study (Project VOW)
Source: Soc Psychiatry Psychiatr Epidemiol. 2026 Aug 28;61(12):2593–604. doi: 10.1007/s00127-026-03207-z (PMC13727802; doi:10.1007/s00127-026-03207-z)
Supplement: Supplementary file 1 [file 127_2026_3207_MOESM1_ESM.docx]

Supplemental Table. *Additional Socio-Demographic Characteristics of the VOW Cohort by Mental Health Status, n (%)*

|  | **Full Sample** | **No MHP** | **Unrecognized MHP** | **Recognized MHP/No DX** | **Recognized MHP/DX** | **Unrecognized DX** |
| --- | --- | --- | --- | --- | --- | --- |
|  | N = 4719 | N = 608 (12.9%) | N = 91 (1.9%) | N = 1417 (30.0%) | N = 2566 (54.4%) | N = 37 (0.8%) |
| **Highest Education Completed** |  |  |  |  |  |  |
| Some high school | 424 (9.00%) | 53 (8.75%) | 17 (18.68%) | 128 (9.03%) | 221 (8.64%) | 5 (13.51%) |
| High school diploma / GED | 1292 (27.44%) | 191 (31.52%) | 39 (42.86%) | 418 (29.50%) | 630 (24.63%) | 14 (37.84%) |
| Vocational/technical training | 51 (1.08%) | 6 (0.99%) | 0 (0.00%) | 13 (0.92%) | 32 (1.25%) | 0 (0.00%) |
| Some college credit | 2472 (52.50%) | 293 (48.35%) | 29 (31.87%) | 720 (50.81%) | 1417 (55.39%) | 13 (35.14%) |
| Associates | 307 (6.52%) | 38 (6.27%) | 5 (5.49%) | 90 (6.35%) | 169 (6.61%) | 5 (13.51%) |
| Bachelors | 149 (3.16%) | 22 (3.63%) | 1 (1.10%) | 44 (3.11%) | 82 (3.21%) | 0 (0.00%) |
| Professional degree (MA, PhD) | 5 (0.11%) | 0 (0.00%) | 0 (0.00%) | 2 (0.14%) | 3 (0.12%) | 0 (0.00%) |
| Other | 9 (0.19%) | 3 (0.50%) | 0 (0.00%) | 2 (0.14%) | 4 (0.16%) | 0 (0.00%) |
| **Post-High School Graduation Plans** |  |  |  |  |  |  |
| Working FT | 24 (5.13%) | 1 (1.45%) | 2 (9.52%) | 7 (5.04%) | 13 (5.56%) | 1 (20.00%) |
| Working PT | 24 (5.13%) | 2 (2.90%) | 1 (4.76%) | 6 (4.32%) | 14 (5.98%) | 1 (20.00%) |
| FT Student-4yr college | 166 (35.47%) | 34 (49.28%) | 7 (33.33%) | 49 (35.25%) | 76 (32.48%) | 0 (0.00%) |
| PT student-4yr college | 5 (1.07%) | 2 (2.90%) | 0 (0.00%) | 0 (0.00%) | 3 (1.28%) | 0 (0.00%) |
| FT student-2yr college | 11 (2.35%) | 4 (5.80%) | 0 (0.00%) | 5 (3.60%) | 2 (0.85%) | 0 (0.00%) |
| PT student-2yr college | 6 (1.28%) | 0 (0.00%) | 1 (4.76%) | 2 (1.44%) | 3 (1.28%) | 0 (0.00%) |
| FT or PT student-online | 1 (0.21%) | 0 (0.00%) | 0 (0.00%) | 0 (0.00%) | 1 (0.43%) | 0 (0.00%) |
| Vocational/Trade School | 3 (0.64%) | 0 (0.00%) | 1 (4.76%) | 0 (0.00%) | 2 (0.85%) | 0 (0.00%) |
| Other | 10 (2.14%) | 1 (1.45%) | 0 (0.00%) | 3 (2.16%) | 6 (2.56%) | 0 (0.00%) |
| Gap Year | 1 (0.21%) | 1 (1.45%) | 0 (0.00%) | 0 (0.00%) | 0 (0.00%) | 0 (0.00%) |
| Multiple Selected | 217 (46.37%) | 24 (34.78%) | 9 (42.86%) | 67 (48.20%) | 114 (48.72%) | 3 (60.00%) |
| **Living Situation** |  |  |  |  |  |  |
| Live at home with parents or other relatives | 2097 (44.52%) | 287 (47.52%) | 54 (60.00%) | 640 (45.23%) | 1095 (42.71%) | 21 (56.76%) |
| Live in a college dorm or apartment | 1575 (33.44%) | 237 (39.24%) | 23 (25.56%) | 487 (34.42%) | 815 (31.79%) | 13 (35.14%) |
| Live on own in an apartment/ townhouse/ house | 232 (4.93%) | 25 (4.14%) | 2 (2.22%) | 59 (4.17%) | 144 (5.62%) | 2 (5.41%) |
| Live with roommates in an apartment/ townhouse/ house | 700 (14.86%) | 50 (8.28%) | 9 (10.00%) | 201 (14.20%) | 439 (17.12%) | 1 (2.70%) |
| Temporarily living in a shelter or unhoused | 16 (0.34%) | 0 (0.00%) | 0 (0.00%) | 3 (0.21%) | 13 (0.51%) | 0 (0.00%) |
| Other living arrangement | 90 (1.91%) | 5 (0.83%) | 2 (2.22%) | 25 (1.77%) | 58 (2.26%) | 0 (0.00%) |
| **Relationship Status** |  |  |  |  |  |  |
| Single, not in a relationship | 1712 (36.50%) | 277 (45.94%) | 45 (50.00%) | 585 (41.46%) | 785 (30.78%) | 20 (54.05%) |
| Single, casual dating | 1024 (21.83%) | 134 (22.22%) | 17 (18.89%) | 285 (20.20%) | 582 (22.82%) | 6 (16.22%) |
| In a relationship, not living together | 1484 (31.64%) | 168 (27.86%) | 22 (24.44%) | 438 (31.04%) | 847 (33.22%) | 9 (24.32%) |
| In a relationship, living together | 401 (8.55%) | 17 (2.82%) | 4 (4.44%) | 82 (5.81%) | 296 (11.61%) | 2 (5.41%) |
| Married/domestic partnership | 68 (1.45%) | 7 (1.16%) | 2 (2.22%) | 21 (1.49%) | 38 (1.49%) | 0 (0.00%) |
| Separated/Divorced | 2 (0.04%) | 0 (0.00%) | 0 (0.00%) | 0 (0.00%) | 2 (0.08%) | 0 (0.00%) |
| **Family Income** |  |  |  |  |  |  |
| No income | 166 (3.53%) | 20 (3.31%) | 8 (8.89%) | 47 (3.33%) | 88 (3.44%) | 3 (8.11%) |
| Less than $15,000 per year | 397 (8.44%) | 37 (6.12%) | 10 (11.11%) | 103 (7.29%) | 245 (9.57%) | 2 (5.41%) |
| $15,000 - $24,999 | 397 (8.44%) | 27 (4.46%) | 8 (8.89%) | 123 (8.71%) | 234 (9.14%) | 5 (13.51%) |
| $25,000 - $34,999 | 378 (8.03%) | 33 (5.45%) | 12 (13.33%) | 123 (8.71%) | 208 (8.12%) | 2 (5.41%) |
| $35,000 - $44,999 | 348 (7.40%) | 42 (6.94%) | 6 (6.67%) | 118 (8.36%) | 180 (7.03%) | 2 (5.41%) |
| $45,000 - $54,999 | 377 (8.01%) | 66 (10.91%) | 5 (5.56%) | 104 (7.37%) | 197 (7.69%) | 5 (13.51%) |
| $55,000 - $74,999 | 593 (12.60%) | 82 (13.55%) | 12 (13.33%) | 200 (14.16%) | 297 (11.60%) | 2 (5.41%) |
| $75,000 - $99,999 | 604 (12.84%) | 87 (14.38%) | 10 (11.11%) | 176 (12.46%) | 327 (12.77%) | 4 (10.81%) |
| $100,000 - $149,999 | 838 (17.81%) | 126 (20.83%) | 11 (12.22%) | 250 (17.71%) | 444 (17.34%) | 7 (18.92%) |
| $150,000 or more per year | 607 (12.90%) | 85 (14.05%) | 8 (8.89%) | 168 (11.90%) | 341 (13.32%) | 5 (13.51%) |
| **Individual Income** |  |  |  |  |  |  |
| No income | 1106 (23.49%) | 142 (23.43%) | 35 (38.46%) | 400 (28.31%) | 517 (20.18%) | 12 (32.43%) |
| Less than $15,000 per year | 2695 (57.23%) | 332 (54.79%) | 39 (42.86%) | 791 (55.98%) | 1515 (59.13%) | 18 (48.65%) |
| $15,000 - $24,999 | 513 (10.89%) | 68 (11.22%) | 11 (12.09%) | 125 (8.85%) | 306 (11.94%) | 3 (8.11%) |
| $25,000 - $34,999 | 194 (4.12%) | 21 (3.47%) | 3 (3.30%) | 46 (3.26%) | 123 (4.80%) | 1 (2.70%) |
| $35,000 - $44,999 | 87 (1.85%) | 12 (1.98%) | 0 (0.00%) | 24 (1.70%) | 49 (1.91%) | 2 (5.41%) |
| $45,000 - $54,999 | 42 (0.89%) | 10 (1.65%) | 2 (2.20%) | 8 (0.57%) | 22 (0.86%) | 0 (0.00%) |
| $55,000 - $74,999 | 30 (0.64%) | 6 (0.99%) | 0 (0.00%) | 12 (0.85%) | 11 (0.43%) | 1 (2.70%) |
| $75,000 - $99,999 | 23 (0.49%) | 9 (1.49%) | 1 (1.10%) | 4 (0.28%) | 9 (0.35%) | 0 (0.00%) |
| $100,000 - $149,999 | 13 (0.28%) | 4 (0.66%) | 0 (0.00%) | 2 (0.14%) | 7 (0.27%) | 0 (0.00%) |
| $150,000 or more per year | 6 (0.13%) | 2 (0.33%) | 0 (0.00%) | 1 (0.07%) | 3 (0.12%) | 0 (0.00%) |
| **Financial Situation: Current** |  |  |  |  |  |  |
| Always stressful | 730 (15.52%) | 23 (3.81%) | 13 (14.61%) | 173 (12.25%) | 515 (20.11%) | 6 (16.22%) |
| Often stressful | 1400 (29.77%) | 100 (16.56%) | 25 (28.09%) | 413 (29.25%) | 855 (33.39%) | 7 (18.92%) |
| Sometimes stressful | 1649 (35.06%) | 265 (43.87%) | 35 (39.33%) | 547 (38.74%) | 787 (30.73%) | 15 (40.54%) |
| Rarely stressful | 749 (15.93%) | 165 (27.32%) | 14 (15.73%) | 234 (16.57%) | 329 (12.85%) | 7 (18.92%) |
| Never stressful | 175 (3.72%) | 51 (8.44%) | 2 (2.25%) | 45 (3.19%) | 75 (2.93%) | 2 (5.41%) |
| **Financial Situation: Childhood** |  |  |  |  |  |  |
| Always stressful | 606 (12.85%) | 22 (3.62%) | 11 (12.09%) | 167 (11.79%) | 403 (15.72%) | 3 (8.11%) |
| Often stressful | 1065 (22.58%) | 82 (13.51%) | 16 (17.58%) | 310 (21.88%) | 646 (25.20%) | 11 (29.73%) |
| Sometimes stressful | 1404 (29.77%) | 217 (35.75%) | 32 (35.16%) | 442 (31.19%) | 706 (27.54%) | 7 (18.92%) |
| Rarely stressful | 1153 (24.45%) | 199 (32.78%) | 22 (24.18%) | 358 (25.26%) | 562 (21.92%) | 12 (32.43%) |
| Never stressful | 488 (10.35%) | 87 (14.33%) | 10 (10.99%) | 140 (9.88%) | 247 (9.63%) | 4 (10.81%) |
| **Food Insecurity: Past year** |  |  |  |  |  |  |
| Never true | 2985 (63.38%) | 483 (79.57%) | 54 (59.34%) | 934 (66.05%) | 1489 (58.14%) | 25 (67.57%) |
| Sometimes true | 1266 (26.88%) | 106 (17.46%) | 29 (31.87%) | 385 (27.23%) | 735 (28.70%) | 11 (29.73%) |
| Often true | 459 (9.75%) | 18 (2.97%) | 8 (8.79%) | 95 (6.72%) | 337 (13.16%) | 1 (2.70%) |
| **Housing instability: Past year** |  |  |  |  |  |  |
| Never true | 3193 (67.78%) | 530 (87.46%) | 59 (64.84%) | 987 (69.80%) | 1588 (61.96%) | 29 (78.38%) |
| Sometimes true | 1108 (23.52%) | 67 (11.06%) | 26 (28.57%) | 329 (23.27%) | 680 (26.53%) | 6 (16.22%) |
| Often true | 410 (8.70%) | 9 (1.49%) | 6 (6.59%) | 98 (6.93%) | 295 (11.51%) | 2 (5.41%) |
| **Health Insurance** | 4152 (88.08%) | 533 (87.81%) | 67 (73.63%) | 1188 (83.90%) | 2330 (90.91%) | 34 (91.89%) |
| **Disability** | 1185 (25.14%) | 31 (5.12%) | 9 (10.00%) | 124 (8.76%) | 1009 (39.35%) | 12 (32.43%) |
| **Religion** |  |  |  |  |  |  |
| Agnostic | 1024 (23.49%) | 56 (9.95%) | 5 (5.81%) | 273 (20.76%) | 685 (28.95%) | 5 (16.67%) |
| Atheist | 733 (16.81%) | 74 (13.14%) | 13 (15.12%) | 211 (16.05%) | 432 (18.26%) | 3 (10.00%) |
| Buddhist | 57 (1.31%) | 4 (0.71%) | 2 (2.33%) | 16 (1.22%) | 34 (1.44%) | 1 (3.33%) |
| Catholic/Roman Catholic | 435 (9.98%) | 126 (22.38%) | 13 (15.12%) | 140 (10.65%) | 154 (6.51%) | 2 (6.67%) |
| Hindu | 41 (0.94%) | 11 (1.95%) | 2 (2.33%) | 15 (1.14%) | 13 (0.55%) | 0 (0.00%) |
| Jewish | 127 (2.91%) | 10 (1.78%) | 0 (0.00%) | 27 (2.05%) | 89 (3.76%) | 1 (3.33%) |
| The Church of Jesus Christ of Latter-day Saints | 91 (2.09%) | 20 (3.55%) | 1 (1.16%) | 35 (2.66%) | 34 (1.44%) | 1 (3.33%) |
| Muslim | 75 (1.72%) | 14 (2.49%) | 5 (5.81%) | 26 (1.98%) | 30 (1.27%) | 0 (0.00%) |
| Orthodox | 31 (0.71%) | 7 (1.24%) | 2 (2.33%) | 7 (0.53%) | 15 (0.63%) | 0 (0.00%) |
| Protestant | 414 (9.50%) | 102 (18.12%) | 9 (10.47%) | 137 (10.42%) | 160 (6.76%) | 6 (20.00%) |
| Nothing in particular | 1053 (24.15%) | 117 (20.78%) | 26 (30.23%) | 343 (26.08%) | 556 (23.50%) | 11 (36.67%) |
| Something else | 279 (6.40%) | 22 (3.91%) | 8 (9.30%) | 85 (6.46%) | 164 (6.93%) | 0 (0.00%) |
| **Political Affiliation** |  |  |  |  |  |  |
| Constitution Party | 1 (0.02%) | 0 (0.00%) | 0 (0.00%) | 1 (0.07%) | 0 (0.00%) | 0 (0.00%) |
| Democrat | 2026 (43.62%) | 260 (43.12%) | 29 (32.22%) | 573 (40.81%) | 1155 (45.98%) | 9 (25.00%) |
| Green Party | 96 (2.07%) | 2 (0.33%) | 1 (1.11%) | 18 (1.28%) | 74 (2.95%) | 1 (2.78%) |
| Independent | 632 (13.61%) | 62 (10.28%) | 10 (11.11%) | 206 (14.67%) | 350 (13.93%) | 4 (11.11%) |
| Libertarian | 121 (2.60%) | 11 (1.82%) | 1 (1.11%) | 36 (2.56%) | 73 (2.91%) | 0 (0.00%) |
| Republican | 263 (5.66%) | 82 (13.60%) | 9 (10.00%) | 80 (5.70%) | 83 (3.30%) | 9 (25.00%) |
| Nothing in particular | 1258 (27.08%) | 182 (30.18%) | 39 (43.33%) | 442 (31.48%) | 582 (23.17%) | 13 (36.11%) |
| Something else | 248 (5.34%) | 4 (0.66%) | 1 (1.11%) | 48 (3.42%) | 195 (7.76%) | 0 (0.00%) |

*Note*. MHP = mental health problem, DX = diagnosis
